# Editorial: Mechanism explorations of enhancing immunotherapeutic sensitivity via mediating immune infiltration and programmed cell death in solid tumor microenvironment

**DOI:** 10.3389/fimmu.2025.1559657

**Published:** 2025-01-31

**Authors:** Fang Zheng, Zhiliang Li, Ruoyu Weng, Baochi Ou, Tao Zhang, Ren Zhao, Haoran Feng

**Affiliations:** ^1^ Department of General Surgery, Ruijin Hospital, Shanghai Jiao Tong University School of Medicine, Shanghai, China; ^2^ Shanghai Institute of Digestive Surgery, Ruijin Hospital, Shanghai Jiao Tong University School of Medicine, Shanghai, China; ^3^ Institute of Medical Robotics, Ruijin Hospital, Shanghai Jiao Tong University School of Medicine, Shanghai, China; ^4^ Department of Surgery, First Affiliated Hospital, Anhui Medical University, Hefei, China; ^5^ Human Oncology and Pathogenesis Program (HOPP), Memorial Sloan Kettering Cancer Center (MSK), New York, NY, United States

**Keywords:** immunotherapy, immune infiltration, programmed cell death, tumor microenvironment, solid cancer

Immunotherapy has become a research hotspot in the field of cancer therapy in recent years. As a therapy which works by activating and regulating the immune system to recognize and attack tumor cells, it shows significant clinical efficacy especially in some refractory tumors and results in fewer side effects. Despite its potential, immunotherapy still faces several challenges in clinical practice, with one of the widely focused issues being the individual variability of patient response (1). Tumor immune microenvironment (TIME) in solid tumor could be classified into two main types as either “high immunogenicity” or “low immunogenicity”, reflecting the immune infiltration pattern of tumor. The former ones typically exhibit better response to immunotherapy while the latter shows the opposite (2, 3). Therefore, regulating tumor immune infiltration to convert the TIME from “cold” to “hot” is considered a promising approach to enhance the efficacy of immunotherapy (4–6).

Checkpoint blockage therapy targeting PD-1/PD-L1 is a significant advancement in the field of cancer immunotherapy in recent years. However, multiple post-translational modifications have been found as protective factors of PD-1/PD-L1, contributing to the stability of protein and the prevention of protein degradation (7). Wang et al. reported the therapeutic enhancement mechanism of an active component in ginsenosides named Rg3 in non-small-cell lung cancer (NSCLC), which could upregulate PD-L1 N-glycosylation. It is noted that Rg3 strengthened the anti-tumor ability of T cells and augmented immune cytotoxicity of CD8^+^T cells. Based on their findings, the combination of Rg3 and immunotherapy is hopeful to enhance the anti-tumor immune response and benefit patients with NSCLC or other solid tumors. Some tumors are inherently resistant to immunotherapies targeting various popular checkpoints, and it is crucial to develop new specific therapeutic targets for these tumors. Tang et al. summarized recent studies about the utilization of single-cell RNA sequencing (scRNA-seq) in uveal melanoma (UM). The analyzed results indicate the presence of intratumoral heterogeneity (ITH), helping tumors cope with challenges. Certain researchers tended to reanalyze existing public data of scRNA-seq to develop advanced predictive models and identify biomarkers for prognosis prediction. In addition, scRNA-seq provided thorough exploration of the complex TME landscape, facilitating potential immunotherapy targets of UM such as LAG-3 for ICIs or specific markers of tumor-associated macrophages (TAMs). Even in the cancers with widespread use of immunotherapy, treatment efficacy varies between patients. Tuersun et al. applied scRNA-seq to analyze the features of certain colorectal cancer (CRC) patients who are more beneficial from neoadjuvant immunotherapy. They trained a novel prognosis model based on a risk score composed of 15 chemokines, performing better in predictive ability than traditional TNM staging. The result of scRNA-seq indicated the difference in tumor stemness between high and low response groups at the same time. CXCL10+ M1 macrophages, which enhance effector T cell migration and convert the TIME from “cold” to “hot,” are reasonable predictors of neoadjuvant therapy. Up to the overall level of TME, adjustment of the proportions of immune cell components also relates to the efficacy of immunotherapy. Ahn et al. focused on mitochondria regulation as enhancement of tumor immunotherapy. The hypoxic glycolysis state in TME refers to mitochondrial metabolic reprogramming, destroying the function of anti-tumor immune cells. However, various immunosuppressive cells develop unique metabolic pathways to adapt the circumstances. Consequently, mitochondrial-targeting drugs, such as metformin and statins, in combination with immunotherapy can regulate the energy metabolism within TME and establish an environment conducive to the activation of anti-tumor immune cells. TLS-TIME is a specific subclass of high immunogenicity TIME, which is correlated to positive prognosis in solid tumor mostly. Yang et al. consolidated potential relationship between gut microbiota and tertiary lymphoid structures (TLSs) in TME. Regarded as significant sites for the activation of adaptive immune responses in the tumor periphery, TLSs are commonly observed in chronic inflammation microenvironment, including the persistent chronic inflammatory status induced by gut microbiota. Regulation on gut microbiome is hopeful to become emerging strategies for modulating the TIME mode by inducing mature TLSs in solid cancer.

Beyond the above, programmed cell death (PCD) is also closely related to the outcome of immunotherapy. Serving as a planned cell elimination tightly regulated by intricate molecular pathways, it contributes to maintaining physiological homeostasis. PCD could either be activated by immunogenic factors, or adjuvant anti-tumor immunity (8). It is known as PCD could be induced by chemotherapy and radiation, and their combination with immunotherapy leads to enhanced therapeutic effect. Studies have reported that direct administration of gasdermin agonist or other PCD-inducing agents may improve the efficacy of cancer immunotherapy similarly (9–11).

Exploration of PCD targets and pathways is essential for upgrading current therapeutic approaches and development of novel ones. Huang et al. tracked research hotspots of TGF-β activated kinase 1 (TAK1) by using bibliometric and visualized analysis. TAK1 is involved in the regulation of TNF, IL-1 and TGF-β pathways, which all related to immune response and regulated cell death. Meanwhile, the lack of TAK1 could trigger PANoptosis, a specific inflammatory PCD combining key features of pyroptosis, apoptosis and necroptosis. The involvement of TAK1 in stages of tumorigenesis, progression and treatment renders it critical research value and promising future prospects. Despite theoretical investigation in laboratories, PCD plays a practical role in clinical treatment. Zheng et al. provided a review with the theme of combination of immune checkpoint inhibitors (ICIs) and carbon ion radiotherapy (CIRT) in renal cell carcinoma (RCC). CIRT possesses enhanced physical and biological characters relative to conventional photon radiation, ensuring accurate complete destruction of cancer cells with maximal protection of healthy tissues. With the co-administration of CIRT and ICIs, TIME will be reshaped into a model with enriched immune infiltration. Moreover, CIRT can trigger immunogenic cell death, a type of PCD following with continuous immune response, to recruit immune cells and stimulate the release of pro-inflammatory cytokines.

In conclusion, enhancing sensitivity has emerges as the central topic in the field of immunotherapy therapy recently. The regulation of either immune infiltration or PCD represents a promising strategy. We deeply appreciate the invaluable contributions of all authors, reviewers, and the editorial team throughout the preparation and review of this topic. With the innovation of technology, several novel approaches to enhance immunotherapy have emerged in recent years, such as designing drug delivery systems by nanomaterials and modifying T cells through CRISPR/Cas9 genome editing technology (12, 13). We are optimistic that future progress in this field will provide additional treatment options, enriching the comprehensive treatment plans for cancer patients.
